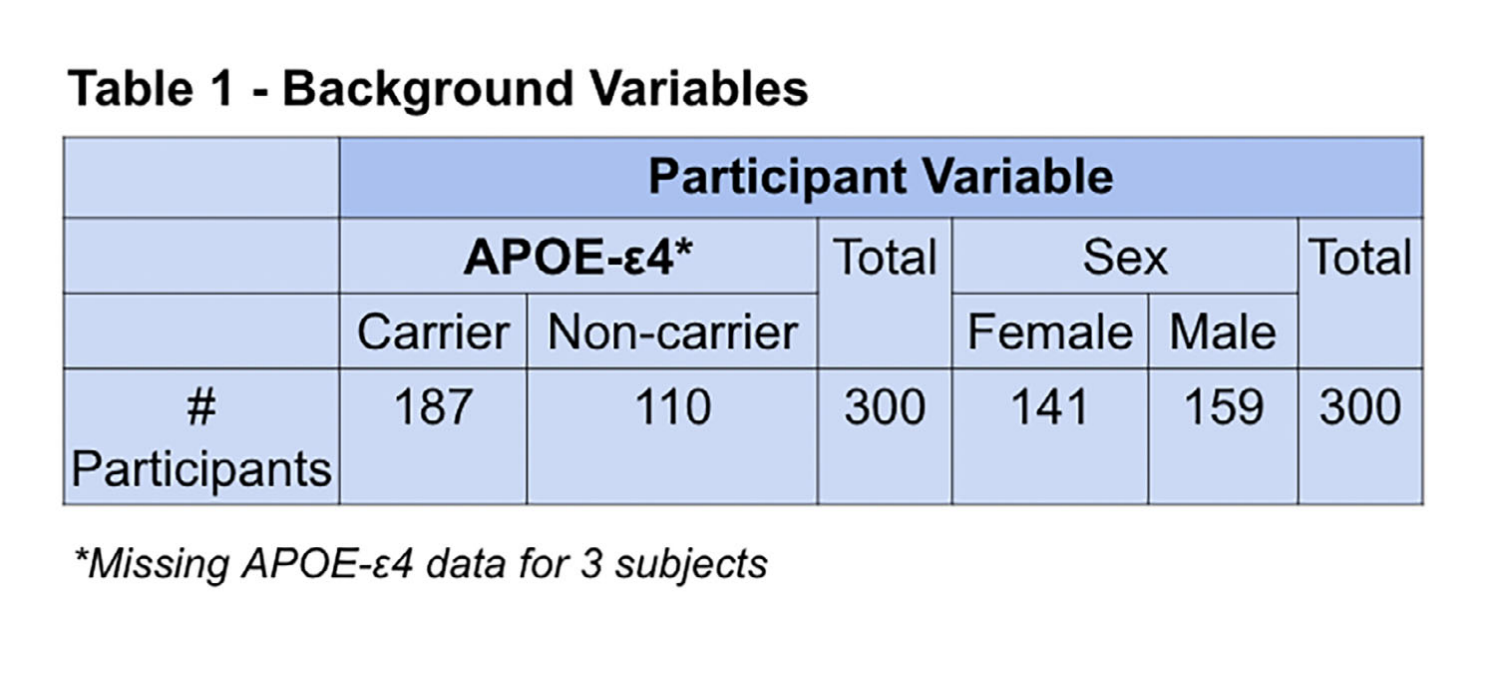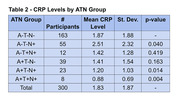# C‐reactive protein as an inflammatory biomarker for early Alzheimer’s Disease pathogenesis

**DOI:** 10.1002/alz.086271

**Published:** 2025-01-03

**Authors:** Aditi Dey, Silke Kern, Ingmar Skoog

**Affiliations:** ^1^ Park Tudor School, Indianapolis, IN USA; ^2^ Department of Psychiatry and Neurochemistry, Institute of Neuroscience and Physiology, University of Gothenburg, Mölndal, Vastra Gotaland Sweden; ^3^ Department of Psychiatry and Neurochemistry, Institute of Neuroscience and Physiology, Sahlgrenska Academy, Centre for Ageing and Health (AgeCap) at the University of Gothenburg, Gothenburg, Sweden., Gothenburg, Vastra Gotaland Sweden

## Abstract

**Background:**

C‐reactive protein (CRP) is an inflammatory biomarker that has been associated with an increased risk of future cognitive decline, alongside other biomarkers such as β‐amyloid (Aβ). We sought to explore the relationship between CRP levels and the amyloid/tau/neurodegeneration (A/T/N) groups in elderly individuals with and without APOE‐ε4.

**Method:**

From 1203 participants of the Gothenburg H70 Birth Cohort study, born in 1944, plasma CRP levels were collected among 300 participants (159 men & 141 women) who did not have dementia. Cerebrospinal fluid (CSF) levels were taken to create ATN groups and explore their correlation with plasma CRP levels. A linear model was created to analyze mean CRP levels in comparison to varying ATN groups. Additionally, the relationship between CRP levels and participant MMSE scores was analyzed.

**Result:**

A negative linear trend was found (p‐value 0.014) with decreasing CRP (0.52 mg/L) in ATN groups with increasing levels of Aβ and p‐tau, meaning CRP decreased as Aβ and p‐tau crossed their respective concentration thresholds to be considered pathological. The A+T+N+ group had lower mean CRP levels than A‐T‐N‐, while the A‐T‐N+ group had higher CRP levels than A‐T‐N‐. Individuals with low Aβ had low CRP more often than those with normal Aβ. No significant difference was found between the mean CRP levels and participants’ MMSE scores (p‐value 0.4117).

**Conclusion:**

In conclusion, the results from this study indicate an association between decreasing CRP levels and the progression of early AD pathogenesis. Though elevations in CRP may be a negative prognostic marker in other diseases, associated with brain aging and inflammation, the immune system might be altered depending on the stage of the disease with CRP levels elevated decades before disease onset, decreased years before and during the early stages of the disease, then finally increased again in more advanced stages of the disease. No relationship was found between CRP level and MMSE score.